# A third-generation microsatellite-based linkage map of the honey bee, *Apis mellifera*, and its comparison with the sequence-based physical map

**DOI:** 10.1186/gb-2007-8-4-r66

**Published:** 2007-05-21

**Authors:** Michel Solignac, Florence Mougel, Dominique Vautrin, Monique Monnerot, Jean-Marie Cornuet

**Affiliations:** 1 Laboratoire Evolution, Génomes et Spéciation, CNRS, 91198 Gif-sur-Yvette cedex, France.; 2 Université Paris-Sud, 91405 Orsay cedex, France.; 3 Centre de Biologie et de Gestion des Populations, Institut National de la Recherche Agronomique, CS 30016 Montferrier-sur-Lez, F34988 Saint-Gély-du-Fesc, France.

## Abstract

**Background:**

The honey bee is a key model for social behavior and this feature led to the selection of the species for genome sequencing. A genetic map is a necessary companion to the sequence. In addition, because there was originally no physical map for the honey bee genome project, a meiotic map was the only resource for organizing the sequence assembly on the chromosomes.

**Results:**

We present the genetic (meiotic) map here and describe the main features that emerged from comparison with the sequence-based physical map. The genetic map of the honey bee is saturated and the chromosomes are oriented from the centromeric to the telomeric regions. The map is based on 2,008 markers and is about 40 Morgans (M) long, resulting in a marker density of one every 2.05 centiMorgans (cM). For the 186 megabases (Mb) of the genome mapped and assembled, this corresponds to a very high average recombination rate of 22.04 cM/Mb. Honey bee meiosis shows a relatively homogeneous recombination rate along and across chromosomes, as well as within and between individuals. Interference is higher than inferred from the Kosambi function of distance. In addition, numerous recombination hotspots are dispersed over the genome.

**Conclusion:**

The very large genetic length of the honey bee genome, its small physical size and an almost complete genome sequence with a relatively low number of genes suggest a very promising future for association mapping in the honey bee, particularly as the existence of haploid males allows easy bulk segregant analysis.

## Abstract 2

The meiotic map of the honey bee is presented, including the main features that emerged from comparisons with the sequence-based physical map. The map is based on 2,008 markers and is about 40 M long, corresponding to a recombination rate of 22 cM/Mb.

## Background

A detailed genetic map is the necessary complement to the sequence in a genome project. Until now, the genetic map pre-dated any genome sequencing, sometimes by many years. For the honey bee, *Apis mellifera *L., whose genome sequence has recently been published [1], only preliminary maps were available at the beginning of the genome project: a random amplification of polymorphic DNA (RAPD) map [2] and a microsatellite map [3]. The improvement of the latter progressed in close relationship with the assembly of the genome sequence, the sequence being a source of markers for mapping and the map providing a framework to set up the assemblies. The simultaneous availability of genetic and physical data also provided the opportunity for mutual quality control and to reach a quasi-colinearity of markers on the two constructions [4]. Here we describe the linkage map of the honey bee and its anchorage on the sequenced-based physical map. We also draw out the emerging properties of the honey bee meioses and compare them to those of some model species. On a large scale, the cumulative genetic distance is close to a linear function of the physical distance, the recombination rate is homogeneous for almost all chromosomes, and the number of recombination events exhibits a minimum variance for individual meioses (close to the stochastic variance). These features do not preclude noticeable positive interference or the existence of a lot of recombination hotspots.

## Results and discussion

### Linkage map

This linkage map, AmelMap3, is based on the segregation of 2,008 markers genotyped in the worker progeny of two queens (B and V, 92 and 95 workers respectively): 1,880 markers for queen B, 662 for queen V, and 534 common markers. Three maps were calculated: the progenies of the two queens taken separately and together (maps B, V, and combined). They were all saturated and as the order of markers in common was the same in maps taken pairwise, the combined map will be the only one considered here (see Additional data files 1 and 2 for the original data used to perform this analysis). The centromeric regions were genetically mapped using half-tetrad analysis of parthenogenetic Cape bees [5] (see Materials and methods). They map in the middle of the largest linkage group (chromosome 1, metacentric) and at one extremity of the remaining 15 telocentric chromosomes (Additional data file 1). The location of telomeric sequences at the opposite end [6] and cytogenetic analyses [1] have confirmed this orientation used for assembly version 4.0 of the genome [1].

The genetic length of the 16 linkage groups, based on the Kosambi function of distance, varies from 575.9 to 138.0 centiMorgans (cM) (Additional data file 1 and 2, Table 1). The total length of the map is 4,114.5 cM. The average density of markers is one every 2.05 cM (Table 1) and all genetic distances between adjacent markers are less than 10 cM. The control of genotypes showing single-locus double recombinants was the most useful method to track genotyping errors. By this method, we detected 500 mistyped genotypes out of 227,322: that is, 0.0022 per marker. Correction of errors was useful to reach colinearity between map and sequence [4]. It also resulted in a reduction in the length of the map by about 1,000 cM (2 cM per mistyping with 100 individuals).

The length of the honey bee genetic map (41 M) is enormous. It is similar to that of the human female genetic map [7] (45 M) despite a genome size 1/10 that of humans (236 Mb [1] versus 2,910 Mb [8]). In *Drosophila melanogaster*, which has a similar genome size to the honey bee (180 Mb), the genetic map is 284.2 cM long, that is 1/15 that of *Apis mellifera*. The genetic map of honey bee chromosome 1 alone (575.9 cM, more than 11 chiasmata on average per meiosis) is more than twice as long as the whole genome of *Drosophila*. Testing the reliability of this value was one of the reasons that we did our best to eradicate genotyping errors, which considerably inflate genetic distances [9].

The length of a genetic map reflects, in terms of crossovers, the number of chiasmata that occurred during meiosis. Chiasmata are assumed to be necessary for proper pairing and segregation of homologous chromosomes. A minimum of one chiasma per bivalent (or per chromosome arm) is essential, and this minimum is observed in many organisms. Accordingly, the minimum genetic size of the genome in centiMorgans is on the order of 50 times the haploid number of chromosomes or of the fundamental number (arm number). A direct and general consequence of this is that chiasma frequency is positively correlated with the haploid number of chromosomes [10]. However, for *n *= 16, the honey bee has a genetic size five times this minimum number and the excess of four chiasmata per bivalent needs to be explained by non-mechanical reasons.

Various explanations have been proposed to account for this large genetic size [2,11,12]. One class invokes an increase in recombination to optimize multilocus selection. Hill and Robertson [13] analyzed the process of selection at two linked loci and showed that selection at one locus hindered the probability of fixation of the beneficial allele at the other locus. Otto and Barton [14] showed that when the Hill-Robertson effect is occurring, it increases the chance of fixation of modifiers at intervening loci that enhance recombination (but are otherwise neutral). A corollary is that modifiers of recombination are confined between syntenic loci under selection, whereas a general increase over the whole genome is the central question to be resolved in the honey bee. Because of the general nature of this explanation, it is not clear why such increased recombination should be observed in the honey bee and not in many other organisms.

Another possible reason is the male haploidy observed in species of Hymenoptera. Deleterious mutations, not protected by dominance, are expressed in haploid males. A high recombination rate may help to remove deleterious genes [15]. Hunt and Page [2] have suggested this idea, which was later criticized by Gadau *et al*. [11] on the grounds that high and low genetic sizes are observed in the Hymenoptera. This rejection was perhaps too hasty, because the suggestion may be considered in conjunction with the low effective population size of the honey bee (see below).

A high recombination rate in females could also be considered as a compensation mechanism for the absence of recombination in males. In fact, in social Hymenoptera, this is not observed: honey bee queens have multiple mates, which contributes to genetic diversity in worker progeny, whereas the singly mated bumblebee *Bombus terrestris *has lower recombination rates in females [16]. In addition, in *Drosophila *there is no recombination in the male germline but the females exhibit one of the shortest genetic maps in insects.

Another possible related factor could be the influence of effective population sizes. Genetic drift is at a maximum in small populations and generates linkage disequilibrium that has adverse effects on multilocus selection. These effects may be counterbalanced by increased recombination rates [17]. For honey bees, the effective population size is very low, on the order of 500 to 1,000 [18,19], as it only includes sexuals - queens and drones - the majority of individuals being sterile workers.

In line with this last hypothesis, artificial selection experiments (which often retain a low proportion of selected individuals and hence generate intense genetic drift) may promote an increase in recombination [17]. A higher recombination rate has also been observed for farm animals compared to non-domesticated mammals [17]. Increased recombination was also observed as a by-product of directional selection in several regions of the genome of *D. melanogaster *[20]. However, in the honey bee, despite its 'domestic' status, artificial selection remains very marginal and probably does not reinforce drift, mainly because of its small 'natural' effective population size.

Another class of explanations is related to the enhancement of genotypic diversity, in relation to social life and other biological traits. For instance, it has been shown that the length of a generation is positively correlated to recombination rate [21]. This 'Red Queen hypothesis' states that in long-lived organisms the environmental conditions change substantially between generations. Highly recombinant progeny will exhibit a great variety of genetic profiles, some of them more fitted to these new conditions (including resistance to short-lived parasites). Generations in the honey bee are rather long for an insect (about two years). This hypothesis may be retained, but one has to note that conditions of life in relatively stable and regulated nests are probably buffered against the environmental changes. Another hypothesis, included in the preceding one, states that high genetic diversity allows resistance to parasite load (to which social species are particularly exposed), but this has been criticized [11].

Besides the Red Queen hypothesis, Burt and Bell [21] also tested the 'tangled bank hypothesis'. This alternative theory states that sex and recombination function to diversify the progeny from each other, thus reducing competition between them. On the basis of extensive analysis of mammalian genomes, these authors rejected the theory that recombination may be related to fecundity. It has to be noted that if the hypothesis had been plausible, it would have been in sharp conflict with kin selection, which, on the contrary, favors cooperation between individuals with similar genotypes.

Finally, division of labor (polyethism) seems to be a promising hypothesis. In the honey bee, separation of tasks is age-dependent but also under the control of genetic factors [22]. Both high female recombination and extreme polyandry generate genotypic diversity within the colonies. This view is in line with the fact that parasitic (solitary) wasps have a shorter genome length and that two other social species, the bumblebee *Bombus terrestris *[16] and the leaf-cutting ant *Acromyrmex echinatior *[23], have intermediate values. Additional empirical data on meiosis in the Hymenoptera and other insects are necessary to test these numerous hypotheses.

### Interference

The estimated length of a genetic map is dependent on the levels of interference in the genome. In the honey bee, the distance function of Haldane, which assumes no interference, provides an estimate of 42.90 Morgans (41.90 M and 42.51 M for queens B and V). That of Kosambi, which assumes an interference with a coincidence level 2*r*, results in 41.15 M (40.09 for B and 39.51 for V), a figure that is 5% lower. The number of recombination events (measured as the average number of allelic phase changes among individuals) provides a third estimate: 39.76 M for B and 38.27 M for V (the means are not significantly different, see below for variances), which is 5% lower than the Kosambi distance function estimate. The reliability of the last estimate is dependent on the probability of undetected single-locus double crossovers. We computed that with probability 0.989 there was no undetected double crossover (UDC) in the progeny of B and 0.804 in the progeny of V. The probability of a single UDC was 0.011 and that of more than one UDC was 6.10^-5 ^in the B progeny and 0.176 and 0.021, respectively, for the progeny of V. Consequently, the true value is probably closer to 39 than 41 M and the positive interference higher than that inferred by the Kosambi function, as in human [24] and mouse [25].

**Figure 1:**
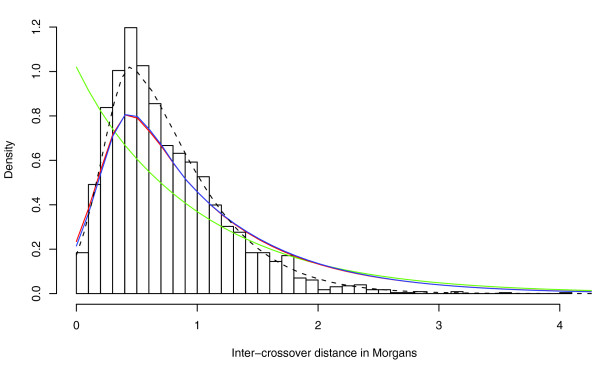
Observed distribution of inter-crossover distances compared to theoretical gamma distributions corresponding to the Haldane and Kosambi map functions. Inter-crossover distances are shown in the histogram; Haldane map functions as the green line and Kosambi map functions as the red line. The dashed black line represent the gamma function fitted to the observed distribution through a maximum likelihood approach. The blue line is the gamma(ν, 2ν) distribution fitted to the observed data. The blue and red lines are almost coincident.

Most map functions can be viewed as related to a stationary renewal chiasma process [26] whose density is well approximated by a gamma distribution. Haldane and Kosambi map functions correspond to renewal processes with densities approximated by gamma(2,1) and gamma(5.2, 2.6). The observed distribution of inter-crossover distances in the honey bee progenies, represented by the histogram in Figure 1 (together with the two gamma distributions of the Haldane and Kosambi map functions) is well approximated by a gamma(3.22, 2.41) (dashed black line) whose mode is higher than for Kosambi, hence corresponding to a higher interference.

**Figure 2:**
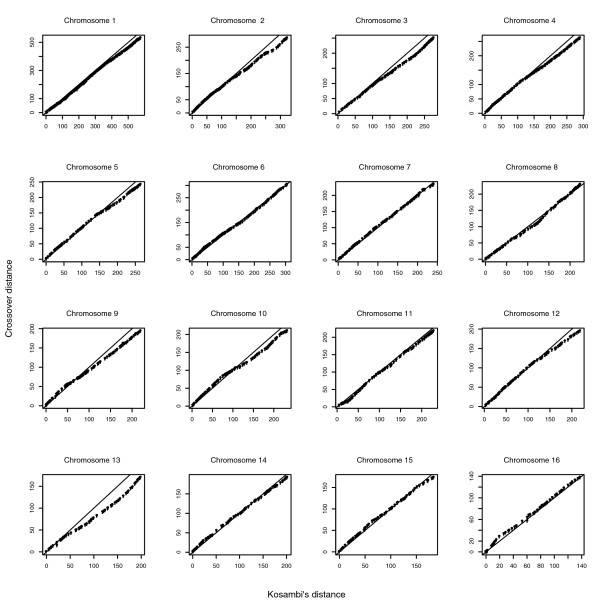
Recombination distance plotted over the Kosambi distance for chromosomes 1 to 16. The recombination distance is directly deduced from allelic phase changes on the map. Note that for chromosome 7 the individual points are superimposed on the diagonal (Kosambi on Kosambi), for chromosome 13 the recombination rate decreases progressively in the distal half, and for chromosome 8 the decrease is central.

**Figure 3:**
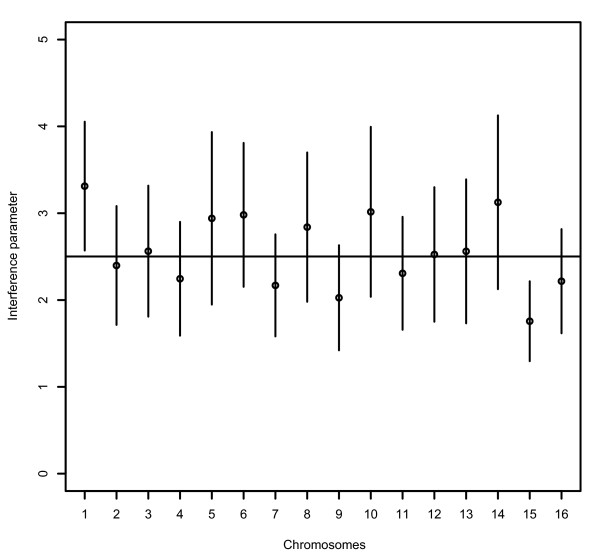
Estimates and confidence intervals of the interference parameter ν from the gamma distribution for the 16 honey bee chromosomes. The estimate for each chromosome is shown as a circle. Confidence intervals (vertical lines) are twice the estimated standard error. The horizontal line is the estimate obtained with all chromosomes.

The cumulative percentages of recombination as a function of the Kosambi distance are plotted in Figure 2. This pattern suggests that interference is correctly inferred by the Kosambi function on the proximal half of most of the chromosomal arms and underestimated for some of them in the distal half. The value of the gamma shape parameter, as an indicator of interference, is given in Table 1 for each chromosome and graphically in Figure 3. The longer chromosomes show a higher level of interference (*r *= 0.56, significant at the 5% level). This relationship is observed whatever the reference used (genetic maps of the two queens or the physical map) and this result is in line with most previous observations (for instance yeast [27] and humans [28]) but contrary to the mouse genome [25].

### Recombination rate

Genetic distances may also be considered in relation to physical lengths, their ratio being the recombination rate. In the honey bee, the conjunction of the large size of the genetic map and the small DNA content produces an extraordinarily high ratio, which averages 22.04 cM/Mb for the assembled part of the genome. In addition, the recombination rate is very similar for all chromosomes. It varies from 17.91 (chromosome 11) to 26.55 (chromosome 4); if these two chromosomes are excluded, the range for the 14 remaining ones becomes as small as 20.33-23.62 and the observed differences are not statistically significant (Table 1, χ132
 MathType@MTEF@5@5@+=feaafiart1ev1aaatCvAUfeBSjuyZL2yd9gzLbvyNv2Caerbhv2BYDwAHbqedmvETj2BSbqee0evGueE0jxyaibaiKI8=vI8tuQ8FMI8Gi=hEeeu0xXdbba9frFj0=OqFfea0dXdd9vqai=hGuQ8kuc9pgc9s8qqaq=dirpe0xb9q8qiLsFr0=vr0=vr0dc8meaabaqaciGacaGaaeqabaqadeqadaaakeaacqaHhpWydaqhaaWcbaGaaGymaiaaiodaaeaacaaIYaaaaaaa@3745@ = 14.73, *P *= 0.32). In addition, the number of crossovers per chromosome is not significantly different in the progenies of the two queens (χ152
 MathType@MTEF@5@5@+=feaafiart1ev1aaatCvAUfeBSjuyZL2yd9gzLbvyNv2Caerbhv2BYDwAHbqedmvETj2BSbqee0evGueE0jxyaibaiKI8=vI8tuQ8FMI8Gi=hEeeu0xXdbba9frFj0=OqFfea0dXdd9vqai=hGuQ8kuc9pgc9s8qqaq=dirpe0xb9q8qiLsFr0=vr0=vr0dc8meaabaqaciGacaGaaeqabaqadeqadaaakeaaiiGacqWFhpWydaqhaaWcbaGaaGymaiaaiwdaaeaacaaIYaaaaaaa@374E@ = 15.69, *P *= 0.40).

In many species there is a large variation in the recombination rate among chromosomes and a general tendency for the smallest chromosomes to recombine more than the large ones. In mammals, this negative correlation is strong for humans but marginal for rodents [29]. The recombination rates are 0.44 to 1.19 for the rat (genomic average 0.60 cM/Mb), 0.44 to 1.05 for the mouse (average 0.56) and 1.07 to 2.10 for human (average 1.26), that is, a twofold variation. An extreme situation exists in the chicken between macro- and microchromosomes, where the range is 2 to 21 cM/Mb [30]. The relationship between recombination rate and chromosome size may be interpreted as the by-product of the requirement for all chromosomes, whatever their size, to make at least one chiasma and this minimum may be driven by chiasma interference [25]. In honey bees, no such tendency is observed and all chromosomes show a very similar recombination rate.

**Figure 4:**
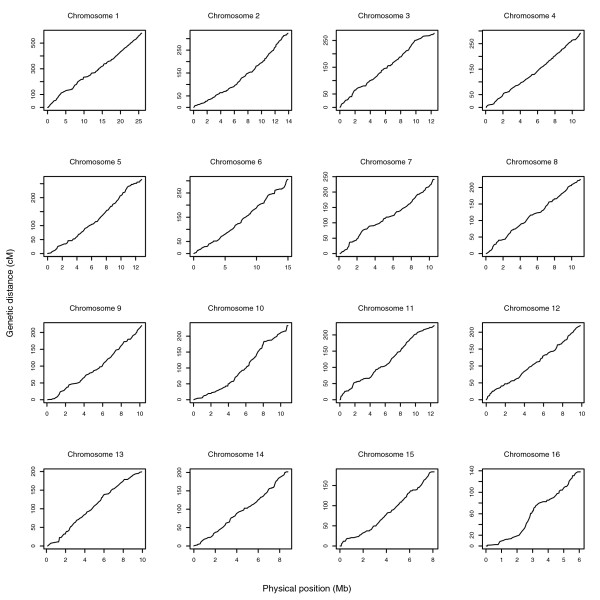
Marey maps showing genetic distance plotted against physical position. The general tendency is close to a straight line. Chromosome 4 is the best example of this linearity. Chromosome 1 shows that the centromeric region does not affect the linear relation (see text for discussion). Chromosomes 10 and 16 show the greatest irregularities. Gaps between scaffolds have been ignored (that is, set at 0 kb) but the graphs obtained fixing the gaps to 50 kb are indistinguishable from these ones.

At a smaller scale, along the arms of a chromosome, a simple way to analyze the possible variations in the recombination rate is to consider the so-called Marey maps [31] (Figure 4). In these graphs, the cumulative genetic distances (here Kosambi, in cM) are plotted against the physical distances (in Mb). Their construction implies some assumptions on the size of sequence and clone gaps remaining in the assembly between adjacent disjoint scaffolds. Superscaffolding (a process that uses all available sources of evidence for designing novel connections or joins between mapped scaffolds) allowed the reduction of 139 scaffolds to 25 superscaffolds by adding only 2.5 Mb (5.5% increase) to the five smallest elements (chromosomes 12 to 16) [32]. This represents an average length for the inter-scaffold gaps of only 21.9 kb in assembly version 4.0. Regardless of the assumption chosen, the picture is robust and is not modified whether the gaps are fixed at 50 kb or are ignored, as they are in Figure 1.

**Figure 5:**
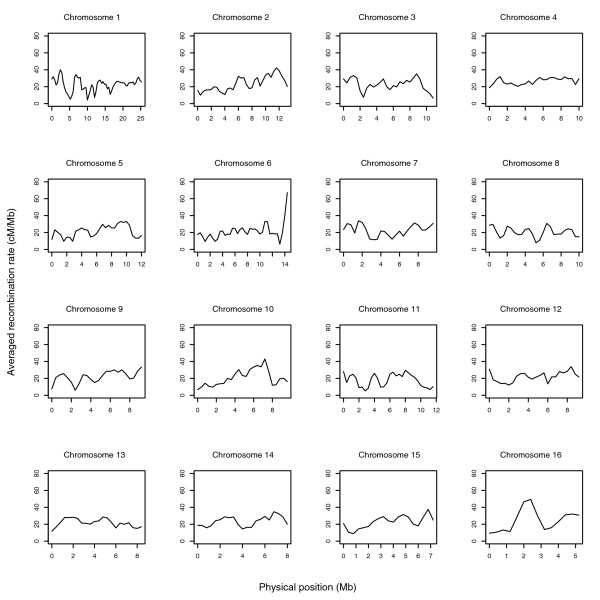
Recombination rate as a function of physical position on the 16 honey bee chromosomes. Recombination rate is the ratio of the genetic distance and the physical distance. A sliding window of 1 Mb and a shift of 0.4 Mb between window centers were used.

Most of the honey bee chromosomes show a linear relationship between cM and Mb and the linearity is almost perfect for chromosome 4 and others. For chromosome 1, the centromeric region is in the middle of the line and there is no evidence of a relaxed recombination rate at this level. It should, however, be noted that pericentromeric sequences (of unknown length) are lacking in the assembly (as for the other species) and their addition could create a plateau. Nevertheless, the proximity of the centromere seems to have little, if any, effect (Figure 4). The Marey maps are, however, monotonic by nature, and local variations of recombination rates may be masked on these graphs. Consequently, we have analyzed the variations of recombination rate at the megabase level using windows of 1 Mb (Figure 5). Similar analyses in mammalian genomes used windows of 1, 3, 5, or 10 Mb [29,33,34], but as the genome of the honey bee is 10 times smaller than mammalian genomes, the smallest value is the most appropriate. The observed range is from 4.2 (in chromosome 1) to 42.2 cM/Mb (in chromosome 2), that is, a 10-fold variation. The telomeric end of chromosome 6 shows a higher value (67.2), which is not considered because it is based on a truncated terminal window. In the mouse, the use of a 1 Mb window resulted in a recombination rate ranging from 0 to 6 cM/Mb, but these values are difficult to compare with the honey bee because of the null value that persists with 10 Mb windows [34]. In the human genome, Yu *et al*. [33] have defined 'deserts' and 'jungles' as regions showing recombination rates differing by more than an order of magnitude, the deserts being below 0.3 cM/Mb and the jungles above 3 cM/Mb. In the honey bee all values are precisely included within the ratio 1:10 and consequently there is no strong evidence for extreme recombination rates and jungles and deserts in its genome. It has, however, to be remarked that if the results depend on the window width and on its relationship to the average size of the physical region encompassing a constant recombination rate (if any) in the genome, they also depend on the absolute number of crossovers. In that respect, the number of crossovers is 10 times higher per physical unit in the honey bee and hence, for the same physical window, the statistical variance (but not necessarily the biological one) is expected to be lower.

Moreover, trends of variation that should be specific to particular regions of the chromosome arms do not appear on the graphs of Figures 4 and 5; the moderate irregularities are rather uneven and do not show general tendencies. The telomeres, which are reached both by the sequence and the map, show no obvious effect (Figure 4). The centromeric side is more questionable. Pericentromeric DNA sequences have not been scaffolded and all the unknown scaffolds that have been mapped in this region belong to the euchromatic part of the genome [1]. An increase in A+T content suggests, however, that heterochromatic regions are close. The proximity of heterochromatin seems to have no obvious effect on the recombination rate on the adjacent euchromatin.

In contrast, gradients of recombination have been observed along chromosomes in many other species. The worm *Caenorhabditis elegans *is atypical but interesting in this respect. Its complement consists of six chromosomes of about the same size, and each pair experiences a single crossover (absolute positive chiasma interference). Most crossovers occur in noncoding terminal regions, the central cluster of genes showing lower-than-average rates of recombination. This pattern contrasts with other species, where recombination occurs mainly in gene-rich regions [35]. In addition, the relationship with the centromere is not relevant, because worm chromosomes do not have localized centromeres. In females of *D. melanogaster*, the metacentric chromosomes 2 and 3 have higher rates of recombination in the terminal parts of the arms, the large pericentromeric region being rarely recombined [36]. The same tendency is also found in humans (where it is more pronounced in female than in male meioses). The increase in recombination rate at telomeric ends is stronger in humans than in rodents [29,36]. The distribution of recombination in relation to properties of chromosomes (G+C-content, gene characteristics, nucleotide polymorphism, repeated sequences) has been analyzed elsewhere [12].

**Figure 6:**
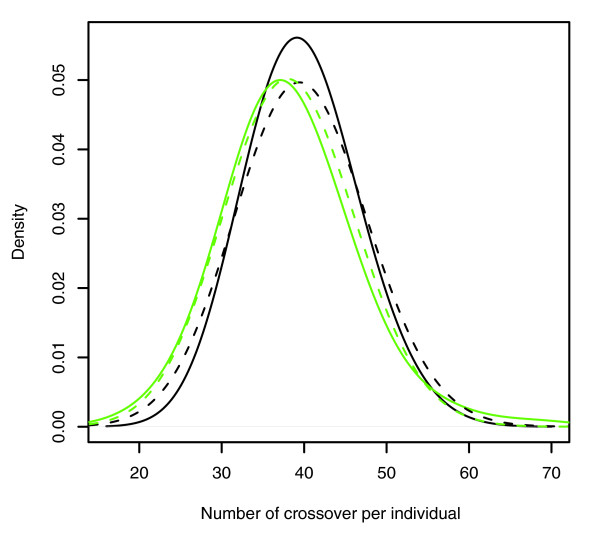
Distribution of number of recombination events per individual. Smoothed distributions of the number of recombination events per individual (solid lines) and the corresponding Poisson distributions (dashed lines) with parameter lambda equal to the average of the observed distribution (lambda = 39.76 for queen B, in black, and lambda = 38.27 for queen V, in green).

The honey bee queen meiosis exhibits several features between and along chromosomes, meioses, and individuals, that denote a tendency towards a strong homogeneity not usually observed in the other species. The two queens show no difference between their recombination rates (the three genetic sizes given above with three different levels of interference differ only by 1.4%, 1.5% and 3.8%). The variances of the number of crossovers for the meioses of a given queen (25.76 for queen B and 53.98 for queen V) are close to the means (39.76 and 38.27 respectively, see Interference section) and thus the distribution is well approximated by a Poisson law (Figure 6). This strongly suggests that the variability is purely stochastic. In other words, this means that crossovers have a fixed probability of occurring at every meiosis (which implies a form of regulation), and that the observed deviations are attributable only to chance. In contrast, in humans there is a high variability in crossovers both within and among individuals [7,37], and for foci numbers in pachytene oocytes [38] and spermatocytes [39]. Moreover, most of the honey bee chromosomes exhibit the same recombination rate. There is no evidence for a higher recombination rate for small chromosomes, as is observed in numerous species. Along chromosomes, variations in the recombination rate are neither localized nor extreme. This also disagrees with numerous observations of the distribution of crossovers/chiasmata/foci in many other organisms [40,41]. All these features denote that, in spite of a very high level of recombination, the number of crossovers and their distribution is at the intersection of relatively strict control and of modulation by chance.

These characteristics of the queen are in sharp contrast to the uneven meioses of the Cape pseudo-queens (laying workers showing thelytokous parthenogenesis, see Materials and methods) [5]. In the latter, the recombination rate is greatly reduced. Moreover, the reduction factor is heterogeneous across the chromosomes and varies from 6.7 to 16.8. Most of the chromosomes show no crossovers at all, and when a chiasma occurs, a second one is often associated (suggesting a negative interference), which restores heterozygosity if lost distally to the first chiasma. Finally, laying workers show an increasing gradient of recombination rate from centromeres to telomeres. All these deviations are prone to maintain heterozygosity in the progeny of laying workers. With the same genotype, queens and pseudo-queens have resolved quite opposite problems: to create maximum genetic diversity for the former, and to preserve the genetic structure of parthenogenetic mothers in their progeny for the latter.

### Hotspots

**Figure 7:**
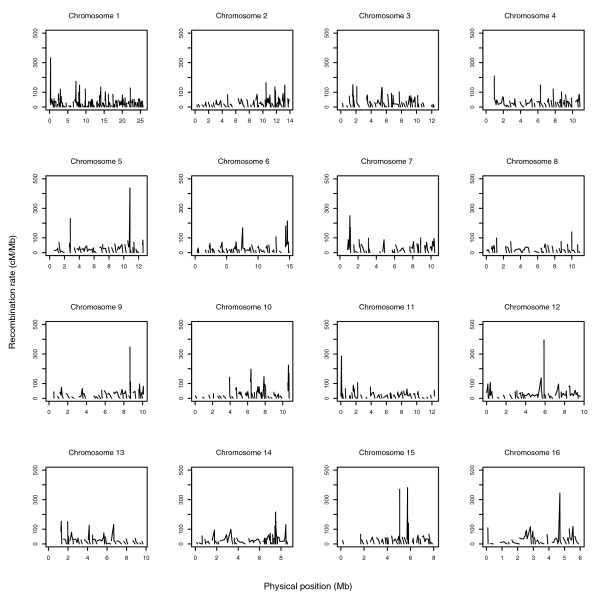
Recombination coldspots and hotspots. The ratio cM/Mb of every interval between consecutive markers within scaffolds was calculated and plotted on the ordinates as a function of the Kosambi cumulative genetic distance between markers on the abscissa. Gaps between scaffolds introduced discontinuities in the graphs. See also Additional data file 1, where yellow sections denote a ratio six times higher than that of the average in the genome (22.04 cM/Mb).

With a recombination rate per physical unit of 22 cM/Mb, the average for the whole genome of the honey bee is 20 times greater than that of humans [42], 1.0 cM/Mb. As the females of the two species have about the same genetic length, this figure is attributable to the smaller C-value (amount of genomic DNA) of the honey bee (236 Mb [1] - but we consider here only the 186 Mb mapped and assembled). In this highly recombining genome, the relative uniformity emphasized above on a large scale is, nevertheless, punctuated by numerous recombination hotspots. A precise analysis of hotspots requires several conditions that are not fulfilled in the construction of a routine genetic map, namely a very high density of markers regularly spaced on the physical scale and large families. Consequently, the conclusions below are provisional. When we plot the recombination rate (cM/Mb) against the physical distance for all chromosomes and all pairs of markers, provided that they are in the same scaffold, very sharp peaks appear (Figure 7). However, if hotspots have the same physical extent in the honey bee as they do in humans (around 1 kb) [43], these peaks do not correspond to the hotspots themselves but to larger regions encompassing them (the average resolution of the map is 2 cM, that is, about 93 kb). In addition, many peaks may correspond to short physical regions that, by chance, have been framed by markers and have shown at least one recombination. For instance, three points have been suppressed (chromosomes 4, 6, and 11). They corresponded to a single recombination event in a very short physical region and provided aberrant values. However, we have checked 106 intervals in the genome that show two to nine crossovers for fewer than 100 individuals and less than 100 kb (average 4.5 recombinations for 45 kb, that is, 100 cM/Mb). They all include potential hotspots. Increasing the density of markers will preserve - or even reinforce - all hotspots detected, and new ones may appear by the subdivision of relatively long regions with intermediate rates.

For further analysis, the honey bee offers a pleasant alternative to single-sperm typing [44] for assessing haplotypes, as haploid drones can be obtained in large numbers from any single queen. The honey bee could thus become the model species for hotspot analysis in invertebrates, the two main biological models (*Drosophila *and *Caenorhabditis*) lacking hotspots [45]. Because coldspots (where recombination is less than expected) and hotspots shape patterns of linkage disequilibrium, they will have their population counterparts (blocks and steps) in haplotype maps, as they do in humans [24,46].

The principal interest of genetic maps is to localize and then identify Mendelian genes or quantitative trait loci (QTLs) in association mapping studies. A long genetic map, as in the honey bee, has both drawbacks and advantages. The identification of candidate regions requires numerous markers for a whole-genome scan but, once identified, it is possible to closely approach the gene of interest with a reasonable number of genotyped individuals. The whole-genome scan may be highly simplified using a bulked segregant analysis [47] with a mixture of DNAs from brother drones or super-sister workers. The second step, the investigation on individual DNAs until null genetic distances are reached, may also be very efficient. Progenies of 300 individuals are easy to get (a queen may lay 2,000 eggs per day) to reach distances of 0.33 cM, which is the domain of single genes, assuming 11,000 genes [1] in the total genome.

## Materials and methods

### Biological features

Reproduction in the honey bee, like other Hymenoptera, is characterized by a haplodiploid system. Differentiation of the two types of females into queens and workers is nutritionally mediated. Both are diploid and produced by sexual reproduction (fertilized oocytes). There is generally a single queen per colony and tens of thousands of sterile workers. Males (drones) are haploid and develop from unfertilized eggs produced by the queen (arrhenotokous parthenogenesis). Male gametogenesis is ameiotic and all spermatozoa produced by a single male have the same genetic profile. Virgin queens mate with a high number of drones (extreme polyandry) in the so-called male congregation areas and store sperm for years. Within a colony, workers produced by the queen are super-sisters (they have the same father) or half-sisters (different fathers).

In addition to arrhenotokous parthenogenesis (drone production), cases of thelytokous (female-producing) parthenogenesis are known. They are occasional in most populations, but are regularly observed in Cape bees, *Apis mellifera capensis *(see also below). In queenless colonies, workers (called pseudo-queens or laying workers) may lay unfertilized eggs that develop into (diploid) females. Diploidization follows a central fusion, that is, the fusion of two of the four products of the meiosis (the availability of two chromatids from the same meiosis in the eggs allows the so-called half-tetrad analysis) that have a central position on the spindles and thus whose centromeres were separated at the first division. In the absence of recombination, this fusion, which follows pre-reduction, restores the heterozygosity of the mother. When a chiasma occurs, the allelic phase is modified, distally to the chiasma, on two chromatids. Depending on the chromatids that occupy the central position, heterozygosity of markers is restored in half of the cases (fusion of two non-recombined chromatids or two recombined ones) or homozygosity appears in the other half (one recombined chromatid, the other not). As chiasmata occur at various locations along the chromosomes, progeny show a gradient of homozygosity from centromeric to telomeric regions and thus chromosomes may be oriented. The centromeric region is genetically defined as the cluster of markers that conserve the heterozygosity of the mother, but the centromere itself is not defined [5].

### Crosses and DNA

Two queens (B and V), hybrids between two subspecies (*Apis mellifera ligustica *× *A. m. mellifera*), were obtained through instrumental insemination. They were backcrossed to two drones, one of each subspecies (each family is composed of two subfamilies of super-sisters). The meioses of the queens were analyzed by genotyping 92 B and 95 V workers as well as the grandfathers to get the allelic phase [3].

### Markers

The markers were cloned at the laboratory [48], or prepared from sequences in GenBank [49], from the first reads of the genome [50] and then from the assemblies of the genome (Baylor Human Genome Sequencing Center). For the latter, it became possible, instead of adding markers at random in the map, to chose them in the scaffolds in previous assemblies in order to orient them, reduce long genetic distances and homogenize density, and also to add numerous 'unknown' scaffolds (not yet assembled) to the successive assemblies. All unknown scaffolds longer than 73 kb of the assembly version 2.0 were mapped (15 new ones that appeared in 4.0 were not mapped) and a few shorter ones were mapped for their specific interest (their gene content or superscaffolding assistance). The list of 2,008 mapped markers is available on the genetic map in Additional data file 2 with genetic and physical information.

### Genotyping

PCR was carried out in conditions as previously described [3]. Over time, a variety of *Taq *polymerases and radionuclides (^32^P, ^35^S, and then ^33^P) were used. Most PCR amplifications were multiplexed and controls were done on eight individuals in single amplifications to confirm the identification of the markers on the gels. The total number of markers mapped is 2,008 (227,322 genotypes). A high density has been preferred to a high precision in order to map as many scaffolds as possible for the same genotyping effort. With about 50 (a single subfamily for a single queen) to 200 meioses per marker (two queen progenies genotyped) and most with 100 (only queen B or V progeny), the confidence interval of the genetic distances is large (for example, for a 2 cM interval and 200 meioses, the 95% confidence interval is [0.55, 5.04]). As stated above, centromeric regions were genetically mapped using half-tetrad analysis on thelytokous Cape bees. The rationale was detailed elsewhere [4]; data have been improved since that time by the addition of new loci. Markers were a sub-sample of those used to construct the map.

### Computation of the map

Computations were performed with CarthaGène software [51], version 0.99. With this version, it is not possible to modify the position of a marker or to integrate physical data. This choice allowed for an independent check of colinearity of markers in the map and the sequence, and a reciprocal control of quality. Lod (log odds ratio) scores for the combined map (two families) are all above 3.7 and may reach 55.7. Null lod scores that correspond to adjacent markers genotyped in different families were observed (126 occurrences) and a lod score control was performed with the closest markers genotyped in the same family.

### Detection of errors

We maintained the same policy as in the first generation map to detect possible genotyping errors [3]. Using single-locus double recombinants (SLDRs, that is, two recombination events surrounding a single marker) as controls was particularly efficient [25,37]. The efficiency of the method increases with the density of the map, but it requires several runs of map calculations/controls (after correction new double recombinants may appear) and is dependent on the assembly used, so this control was relegated to the end of map construction. It was first done using the order directly given by the calculated map. When discrepancies with the sequence persisted, the order of markers was modified according to the sequence and the SLDRs that appeared were controlled. Terminal markers showing a recombination with the second marker were also reamplified. After corrections, the rare loci for which the order was not that of the sequence (almost all were adjacent and with short distances) corresponded to markers genotyped in only one or the other queen. These discrepancies were eradicated by genotyping family V for the loci surrounding the inversion (all markers heterozygous in queen B being already genotyped).

### Statistical analyses

A first homogeneity χ^2 ^test was applied to compare the cumulative number of recombinations per chromosome in the two queen progenies (keeping equal progeny sizes). A second homogeneity χ^2 ^test was applied to test whether the ratios of cM/Mb per chromosome were not significantly different across chromosomes. This test compared observed and estimated cumulative numbers of recombinations in the two queen progenies. The estimated numbers of recombinations were computed by multiplying the number of base pairs in each chromosome by the total number of recombinations divided by the total physical length of the genome.

Computations of the gamma(ν, 2ν) distribution fitted to observed data of inter-crossover distances followed the method of Broman and Weber [28]. Direct fit of the gamma distribution used the maximum likelihood method [52]. All these computations were performed in R (v2.0.1).

To compute the probability of 'hidden' double recombinants (HDRs) between two successive markers, we used the following rationale. The probability of an HDR between two successive markers is the probability that the distance between two recombination events is shorter than the distance between the two markers. It is then the integral of the density function of inter-event distance on [0, *d*], *d *being the distance (in Morgans) between the two markers. As shown on Figure 1, the inter-event distance is well approximated by a gamma density function with shape = 2.409 and scale = 3.223. If we assume that there can be no more than one HDR between two successive markers, the probability that there is no HDR between two successive markers is one minus the previously defined probability. For the complete genome, we compute the product of such probabilities over all intervals between adjacent markers.

## Additional data files

The following data are available with the online version of this paper. Additional data file 1 is a PDF file containing a microsatellite-based genetic map of the honey bee, AmelMap3. Additional data file 2 is an Excel file containing the linkage map of the honey bee AmelMap3 with the following entries: marker name, genotyped queen(s), genetic distances and cumulative coordinates with Haldane and Kosambi functions, accession numbers of the markers, scaffold number (assembly 4.0), orientation of the scaffolds, block of non-ordered scaffolds, scaffold size and coordinate of our upper primers on the scaffolds, sequence of the upper and lower primers, and data used for centromere mapping. The meaning of the columns and lines in the Excel file are given in the file itself.

## Supplementary Material

Additional data file 1 A PDF file containing a microsatellite-based genetic map of the honey bee, AmelMap3. Click here for file

Additional data file 2 An Excel file containing the linkage map of the honey bee AmelMap3 with the following entries: marker name, genotyped queen(s), genetic distances and cumulative coordinates with Haldane and Kosambi functions, accession numbers of the markers, scaffold number (assembly 4.0), orientation of the scaffolds, block of non-ordered scaffolds, scaffold size and coordinate of our upper primers on the scaffolds, sequence of the upper and lower primers, and data used for centromere mapping. The meaning of the columns and lines in the Excel file are given in the file itself. Click here for file

## Figures and Tables

**Table 1:** Characteristics of the third-generation linkage map of the honey bee

Linkage group	Number of markers	Genetic length (cM)		Recombinant		Density of markers	Physical map		Recombination rate (cM/Mb)	Interference: gamma shape parameter
							
		Haldane function	Kosambi function	B map	V map		Number of scaffolds	Assembled length (bp)		
LG01	273	600.6	575.9	553.3	543.2	2.11	83	25,834,090	22.29	3.49
LG02	143	335.6	321.9	296.7	305.3	2.25	43	13,972,177	23.04	2.61
LG03	137	288.6	276.9	260.9	265.3	2.02	39	11,721,520	23.62	2.76
LG04	115	302.1	290.9	272.8	260.0	2.53	27	10,956,690	26.55	2.47
LG05	121	278.9	265.3	252.2	253.7	2.19	33	12,900,692	20.56	3.37
LG06	139	318.7	305.7	316.3	266.3	2.20	55	15,039,083	20.33	3.18
LG07	117	246.4	237.9	239.1	205.3	2.03	47	10,548,973	22.55	2.42
LG08	112	233.1	224.3	239.1	189.5	2.00	47	10,889,223	20.60	3.18
LG09	105	229.8	220.3	202.2	202.1	2.10	26	9,832,907	22.40	2.26
LG10	124	241.4	232.5	218.5	226.3	1.88	45	10,442,577	22.26	3.14
LG11	125	233.9	223.4	222.8	208.4	1.79	42	12,471,977	17.91	2.50
LG12	100	228.2	219.3	203.3	220.0	2.19	30	9,859,010	22.24	2.72
LG13	95	206.6	197.7	175.0	192.6	2.08	21	9,266,737	21.33	2.54
LG14	107	208.1	200.5	200	186.3	1.87	25	8,776,661	22.84	3.38
LG15	112	194.3	184.0	180.4	177.9	1.64	42	8,109,687	22.69	1.88
LG16	83	143.7	138.0	143.5	125.3	1.66	21	6,072,872	22.72	2.23
Total	2,008	4,290.0	4,114.5	3,976.1	3,827.4	2.05	626	186,694,876	22.04	2.70

For each linkage group and the whole genome (total) are indicated: the number of markers mapped (1,346 for family B alone, 128 for family V alone, 534 for both); the genetic length of the map: Haldane and Kosambi functions of distance calculated on the combined map (B + V) (the Kosambi function is used for the calculation of the ratios in the other columns); and the number of recombinations for families B and V normalized by progeny numbers; the density of markers (calculation including null distances) - the density is homogeneous for all chromosomes except 15 and 16 which were further worked for superscaffolding assistance (see text), all distances are smaller than 10 cM; the number of scaffolds that were integrated in assembly version 4.0 with AmelMap3 as the framework; the physical length of the scaffolds in assembly 4.0; the recombination rate as a ratio of the genetic length in centimorgans (cM) over the physical length in megabases (Mb). interference: shape parameter of the fitted gamma(ν, 2ν) distribution.

## References

[B1] Honeybee Genome Sequencing Consortium (2006). Insights into social insects from the genome of the honeybee *Apis mellifera*.. Nature.

[B2] Hunt GJ, Page RE (1995). Linkage map of the honey bee, *Apis mellifera*, based on RAPD markers.. Genetics.

[B3] Solignac M, Vautrin D, Baudry E, Mougel F, Loiseau A, Cornuet JM (2004). A microsatellite-based linkage map of the honeybee, *Apis mellifera *L.. Genetics.

[B4] Solignac M, Zhang L, Mougel F, Li B, Vautrin D, Monnerot M, Cornuet JM, Worley KC, Weinstock GM, Gibbs RA (2007). The genome of *Apis mellifera*: dialog between linkage mapping and sequence assembly.. Genome Biol.

[B5] Baudry E, Kryger P, Allsopp M, Koeniger N, Vautrin D, Mougel F, Cornuet JM, Solignac M (2004). Whole-genome scan in thelytokous-laying workers of the Cape honeybee (*Apis mellifera capensis*): central fusion, reduced recombination rates and centromere mapping using half-tetrad analysis.. Genetics.

[B6] Robertson HM, Gordon KH (2006). Canonical TTAGG-repeat telomeres and telomerase in the honey bee, *Apis mellifera*.. Genome Res.

[B7] Kong A, Gudbjartsson DF, Sainz J, Jonsdottir GM, Gudjonsson SA, Richardsson B, Sigurdardottir S, Barnard J, Hallbeck B, Masson G (2002). A high-resolution recombination map of the human genome.. Nat Genet.

[B8] Venter JC, Adams MD, Myers EW, Li PW, Mural RJ, Sutton GG, Smith HO, Yandell M, Evans CA, Holt RA (2001). The sequence of the human genome.. Science.

[B9] Brzustowicz LM, Merette C, Xie X, Townsend L, Gilliam TC, Ott J (1993). Molecular and statistical approaches to the detection and correction of errors in genotype databases.. Am J Hum Genet.

[B10] Imai HT, Wada MY, Hirai H, Matsuda Y, Tsuchiya K (1999). Cytological, genetic and evolutionary functions of chiasmata based on chiasma graph analysis.. J Theor Biol.

[B11] Gadau J, Page RE, Werren JH, Schmid-Hempel P (2000). Genome organization and social evolution in Hymenoptera.. Naturwissenschaften.

[B12] Beye M, Gattermeier I, Hasselmann M, Gempe T, Schioett M, Baines JF, Schlipalius D, Mougel F, Emore C, Rueppell O (2006). Exceptionally high levels of recombination across the honey bee genome.. Genome Res.

[B13] Hill WG, Robertson A (1966). The effect of linkage on limits to artificial selection.. Genet Res.

[B14] Otto SP, Barton NH (1997). The evolution of recombination: removing the limits to natural selection.. Genetics.

[B15] Kondrashov AS (1988). Deleterious mutations and the evolution of sexual reproduction.. Nature.

[B16] Wilfert L, Gadau J, Schmid-Hempel P (2006). A core linkage map of the bumblebee *Bombus terrestris*.. Genome.

[B17] Otto SP, Barton NH (2001). Selection for recombination in small populations.. Evolution Int J Org Evolution.

[B18] Estoup A, Garnery L, Solignac M, Cornuet JM (1995). Microsatellite variation in honey bee (*Apis mellifera *L.) populations: hierarchical genetic structure and test of the infinite allele and stepwise mutation models.. Genetics.

[B19] Baudry E, Solignac M, Garnery L, Gries M, Cornuet JM, Koeniger N (1998). Relatedness among honey bees (*Apis mellifera*) of a drone congregation.. Proc R Soc Lond B.

[B20] Korol AB, Iliadi KG (1994). Increased recombination frequencies resulting from directional selection for geotaxis in *Drosophila*.. Heredity.

[B21] Burt A, Bell G (1987). Mammalian chiasma frequencies as a test of two theories of recombination.. Nature.

[B22] Robinson GE, Grozinger CM, Whitfield CW (2005). Sociogenomics: social life in molecular terms.. Nat Rev Genet.

[B23] Sirvio A, Gadau J, Rueppell O, Lamatsch D, Boomsma JJ, Pamilo P, Page RE (2006). High recombination frequency creates genotypic diversity in colonies of the leaf-cutting ant *Acromyrmex echinatior*.. J Evol Biol.

[B24] Tapper W, Collins A, Gibson J, Maniatis N, Ennis S, Morton NE (2005). A map of the human genome in linkage disequilibrium units.. Proc Natl Acad Sci USA.

[B25] Broman KW, Rowe LB, Churchill GA, Paigen K (2002). Crossover interference in the mouse.. Genetics.

[B26] Zhao H, Speed TP (1996). On genetic map functions.. Genetics.

[B27] Kaback DB, Barber D, Mahon J, Lamb J, You J (1999). Chromosome size-dependent control of meiotic reciprocal recombination in *Saccharomyces cerevisiae*: the role of crossover interference.. Genetics.

[B28] Broman KW, Weber JL (2000). Characterization of human crossover interference.. Am J Hum Genet.

[B29] Jensen-Seaman MI, Furey TS, Payseur BA, Lu Y, Roskin KM, Chen CF, Thomas MA, Haussler D, Jacob HJ (2004). Comparative recombination rates in the rat, mouse, and human genomes.. Genome Res.

[B30] International Chicken Genome Sequencing Consortium (2004). Sequence and comparative analysis of the chicken genome provide unique perspectives on vertebrate evolution.. Nature.

[B31] Chakravarti A (1991). A graphical representation of genetic and physical maps: the Marey map.. Genomics.

[B32] Robertson HM, Reese J, Milshina N, Agarwala R, Solignac M, Walden KK, Elsik C (2007). Manual superscaffolding of honey bee (*Apis mellifera*) chromosomes 12-16: implications for the draft genome assembly version 4, gene annotation, and chromosome structure.. Insect Mol Biol.

[B33] Yu A, Zhao C, Fan Y, Jang W, Mungall AJ, Deloukas P, Olsen A, Doggett NA, Ghebranious N, Borman KW, Weber JL (2001). Comparison of human genetic and sequence-based physical maps.. Nature.

[B34] Shifman S, Bell JT, Copley RR, Taylor MS, Williams RW, Mott R, Flint J (2006). A high-resolution single nucleotide polymorphism genetic map of the mouse genome.. PLoS Biol.

[B35] Barnes TM, Kohara Y, Coulson A, Hekimi S (1995). Meiotic recombination, noncoding DNA and genomic organization in *Caenorhabditis elegans*.. Genetics.

[B36] Nachman MW (2002). Variation in recombination rate across the genome: evidence and implications.. Curr Opin Genet Dev.

[B37] Broman KW, Murray JC, Sheffield VC, White RL, Weber JL (1998). Comprehensive human genetic maps: individual and sex-specific variation in recombination.. Am J Hum Genet.

[B38] Tease C, Hartshorne GM, Hulten MA (2002). Patterns of meiotic recombination in human fetal oocytes.. Am J Hum Genet.

[B39] Lynn A, Koehler KE, Judis L, Chan ER, Cherry JP, Schwartz S, Seftel A, Hunt PA, Hassold TJ (2002). Covariation of synaptonemal complex length and mammalian meiotic exchange rates.. Science.

[B40] Bishop DK, Zickler D (2004). Early decision; meiotic crossover interference prior to stable strand exchange and synapsis.. Cell.

[B41] Kauppi L, Jeffreys AJ, Keeney S (2004). Where the crossovers are: recombination distributions in mammals.. Nat Rev Genet.

[B42] Fearnhead P, Harding RM, Schneider JA, Myers S, Donnelly P (2004). Application of coalescent methods to reveal fine-scale rate variation and recombination hotspots.. Genetics.

[B43] Crawford DC, Bhangale T, Li N, Hellenthal G, Rieder MJ, Nickerson DA, Stephens M (2004). Evidence for substantial fine-scale variation in recombination rates across the human genome.. Nat Genet.

[B44] Cui XF, Li HH, Goradia TM, Lange K, Kazazian HH, Galas D, Arnheim N (1989). Single-sperm typing: determination of genetic distance between the G gamma-globin and parathyroid hormone loci by using the polymerase chain reaction and allele-specific oligomers.. Proc Natl Acad Sci USA.

[B45] Hey J (2004). What's so hot about recombination hotspots.. PLoS Biol.

[B46] Jeffreys AJ, Kauppi L, Neumann R (2001). Intensely punctate meiotic recombination in the class II region of the major histocompatibility complex.. Nat Genet.

[B47] Michelmore RW, Paran I, Kesseli RV (1991). Identification of markers linked to disease-resistance genes by bulked segregant analysis: a rapid method to detect markers in specific genomic regions by using segregating populations.. Proc Natl Acad Sci USA.

[B48] Solignac M, Vautrin D, Loiseau A, Mougel F, Baudry E, Estoup A, Garnery L, Haberl M, Cornuet J-M (2003). Five hundred and fifty microsatellite markers for the study of the honeybee (*Apis mellifera *L.) genome.. Mol Ecol Notes.

[B49] Whitfield CW, Band MR, Bonaldo MF, Kumar CG, Liu L, Pardinas JR, Robertson HM, Soares MB, Robinson GE (2002). Annotated expressed sequence tags and cDNA microarrays for studies of brain and behavior in the honey bee.. Genome Res.

[B50] Tomkins JP, Luo M, Fang GC, Main D, Goicoechea JL, Atkins M, Frisch DA, Page RE, Guzman-Novoa E, Yu Y (2002). New genomic resources for the honey bee (*Apis mellifera *L.): development of a deep-coverage BAC library and a preliminary STC database.. Genet Mol Res.

[B51] Schiex T, Gaspin C (1997). CARTHAGENE: constructing and joining maximum likelihood genetic maps.. Proc Int Conf Intell Syst Mol Biol.

[B52] Evans M, Hastings N, Peacock B (2000). Statistical Distributions.

