# Supplementary material for: A third-generation microsatellite-based linkage map of the honey bee, Apis mellifera, and its comparison with the sequence-based physical map
Source: Genome Biol. 2007 May 21;8(4):R66. doi: 10.1186/gb-2007-8-4-r66 (PMC1896015; doi:10.1186/gb-2007-8-4-r66)
Supplement: Additional data file 1 — A PDF file containing a microsatellite-based genetic map of the honey bee, AmelMap3. [file gb-2007-8-4-r66-S1.pdf]

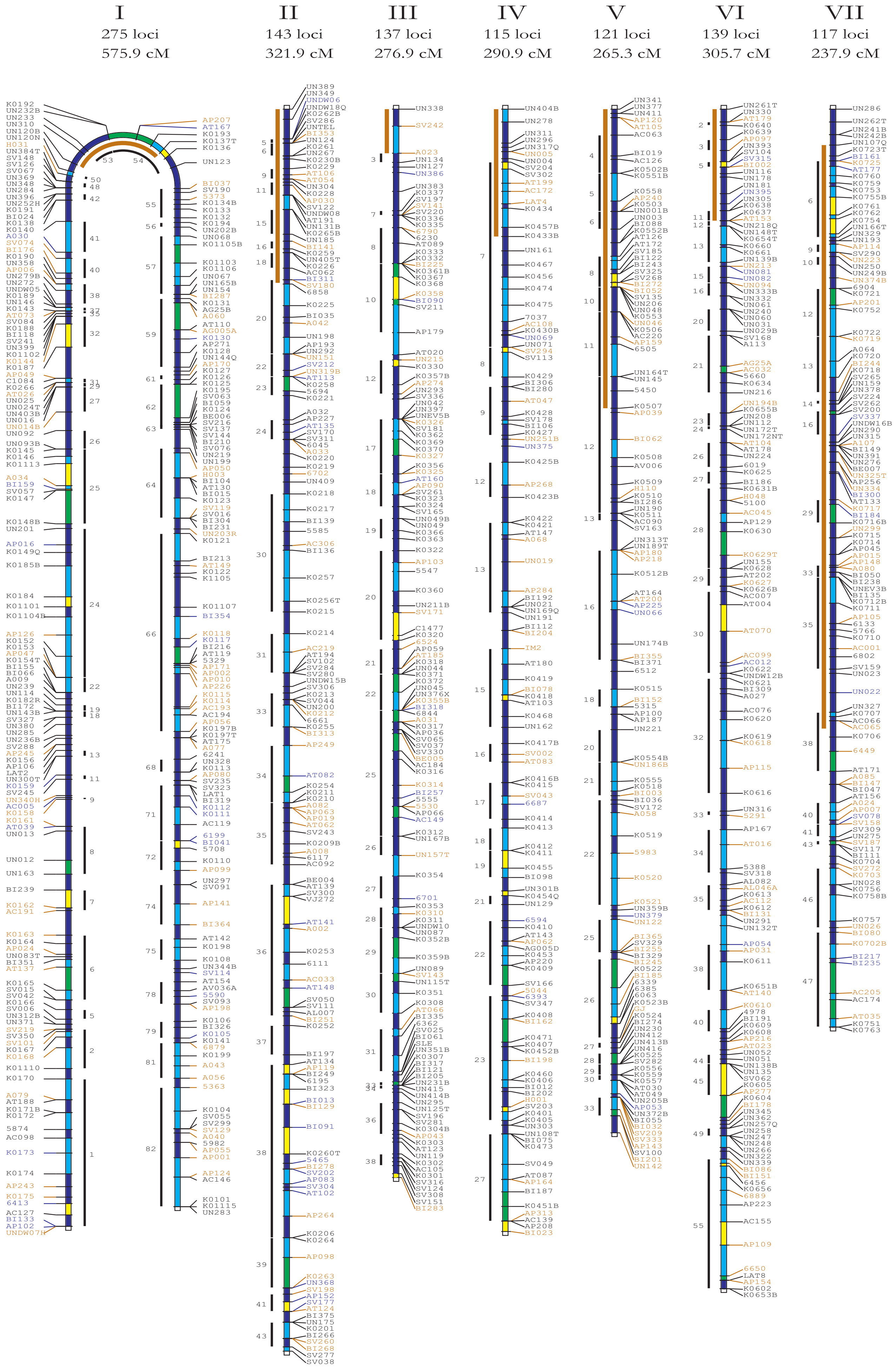
I
275 loci
575.9 cM
II
143 loci
321.9 cM
III
137 loci
276.9 cM
IV
115 loci
290.9 cM
V
121 loci
265.3 cM
VI
139 loci
305.7 cM
VII
117 loci
237.9 cM

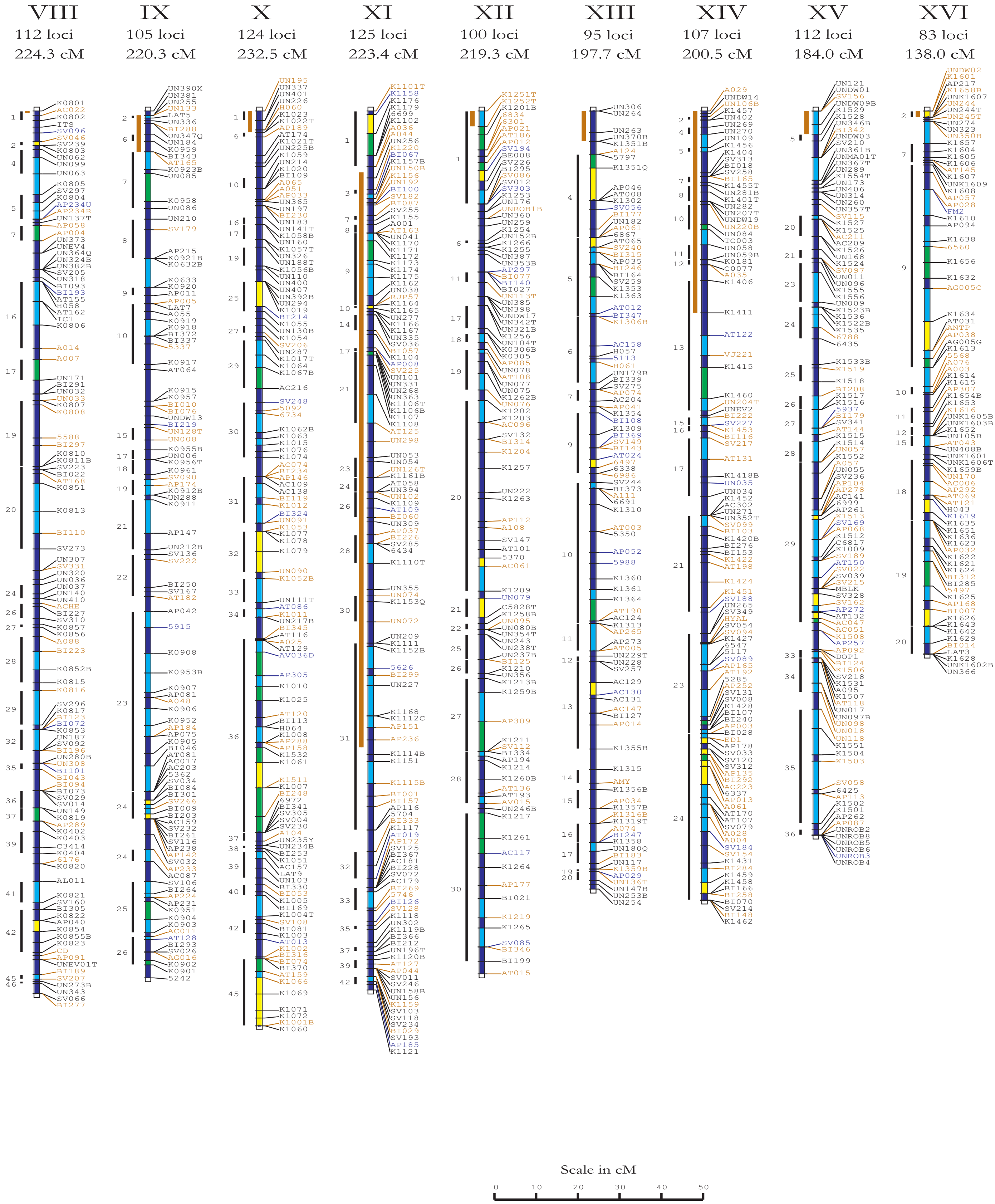

VIII
112 loci
224.3 cM
IX
105 loci
220.3 cM
X
124 loci
232.5 cM
XI
125 loci
223.4 cM
XII
100 loci
219.3 cM
XIII
95 loci
197.7 cM
XIV
107 loci
200.5 cM
XV
112 loci
184.0 cM
XVI
83 loci
138.0 cM
Scale in cM
0
10
20
30
40
50

Additional data file 1

The microsatellite-based genetic map of the honey bee, AmelMap3. The chromosomes have been oriented through half-tetrad analysis of thelytokous Cape bees: centromeric regions, underlined in brown, are at the top. The positions of microsatellite markers on the map have been computed from queen B progeny (brown), queen V progeny (blue), or both progenies (black). The scale in cM corresponds to the Kosambi function of distance. The chromosome number, the numbers of markers and the genetic length of the linkage groups are indicated at the top. The colors on the arms indicate the ratio of the recombination rate over the physical distance: deep blue, 0-35 cM/Mb; light blue, 35-70; green, 70-135; yellow, >135. The longest scaffolds are plotted in black along the arms (the position of short scaffolds may be inferred from the missing numbers) and most are oriented. The length of the lines corresponds to the genetic length rather than the physical length. Another version of the map is available in Additional data file 2.
